# Community based distribution of oral HIV self-testing kits in Zambia: a cluster-randomised trial nested in four HPTN 071 (PopART) intervention communities

**DOI:** 10.1016/S2352-3018(18)30258-3

**Published:** 2018-12-21

**Authors:** Chama Mulubwa, Bernadette Hensen, Mwelwa M Phiri, Kwame Shanaube, Albertus J Schaap, Sian Floyd, Comfort R Phiri, Chiti Bwalya, Virginia Bond, Musonda Simwinga, Lawrence Mwenge, Sarah Fidler, Richard Hayes, Alwyn Mwinga, Helen Ayles

**Affiliations:** a Zambart, University of Zambia, Lusaka, Zambia; b Clinical Research Department, Faculty of Infectious and Tropical Diseases, London School of Hygiene & Tropical Medicine, London, UK; c Department of Infectious Disease Epidemiology, Faculty of Epidemiology and Population Health, London School of Hygiene & Tropical Medicine, London, UK; d Department of Global Health and Development, Faculty of Public Health and Policy, London School of Hygiene & Tropical Medicine, London, UK; e HIV Clinical Trials Unit, Imperial College London, London, UK

## Abstract

**Background:**

The HPTN 071 (PopART) cluster-randomised trial provided door-to-door HIV testing services to a large proportion of individuals residing in 21 intervention communities in Zambia and South Africa from 2014 to 2017 and reached the UNAIDS first 90 target among women in Zambia, yet gaps remained among men and young adults. This cluster-randomised study nested in the HPTN 071 (PopART) trial sought to increase knowledge of HIV status across all groups by offering the choice of oral HIV self-testing in addition to routine door-to-door HIV testing services.

**Methods:**

We nested this cluster-randomised trial in four HTPN 071 (PopART) intervention communities in northern Zambia. 66 zones (clusters) in these communities were randomly allocated (1:1) to either oral HIV self-testing plus routine door-to-door HIV testing services (HIV self-testing group) or the PopART standard of care of door-to-door HIV testing services alone (non- HIV self-testing group) over a 3-month period. All individuals aged 16 years or older were eligible for HIV testing. Randomisation was achieved by randomly selecting one allocation from a list of 10 000 possible allocations during a public ceremony. In HIV self-testing zones, trained lay-counsellors (known as community HIV care providers) visited households and offered eligible individuals the choice of HIV testing using HIV self-testing or routine door-to-door HIV testing services. For individuals aged 18 years or older whose partner was absent during the household visit, an HIV self-test kit could be left for secondary distribution to the absent partner. The primary outcome was knowledge of HIV status (defined as self-reporting HIV positive to the community HIV care providers or accepting an offer of HIV testing services). Outcomes were measured among households that were first visited, and individuals first enumerated as a household member during the HIV self-testing intervention period. We analysed data at the individual level using population-average logistic regression models, accounting for clustering of outcomes by zone, to estimate the effect of the intervention. This trial is registered with ClinicalTrials.gov, number NCT02994329.

**Findings:**

Between Feb 1, and April 30, 2017, the community HIV care providers enumerated 13 267 eligible individuals in the HIV self-testing group and 13 706 in the non-HIV self-testing group. After intervention implementation, 9027 (68%) of 13 267 in the HIV self-testing group had knowledge of HIV status compared with 8952 (65%) of 13 706 in the non-HIV self-testing group (adjusted odds ratio 1·30, 95% CI 1·03–1·65; p=0·03). The effect differed by sex (p_interaction_=0·01). Among men, knowledge of HIV status was higher in the HIV self-testing group than in the non-HIV self-testing group (3843 [60%] of 6368 *vs* 3571 [55%] of 6486; adjusted odds ratio 1·31, 95% CI 1·07–1·60; p=0·01). There was no evidence of a between-group difference among female participants.

**Interpretation:**

Providing a choice of HIV self-testing during delivery of door-to-door HIV testing services increased knowledge of HIV status, driven by an effect among men. Lay counsellors have a vital role to play in adapting HIV self-testing interventions to local context.

**Funding:**

The International Initiative for Impact Evaluation (3ie), the Bill & Melinda Gates Foundation, National Institute of Allergy and Infectious Diseases, National Institute on Drug Abuse, National Institute of Mental Health, and the President's Emergency Plan for AIDS Relief.

## Introduction

Despite widespread availability of facility-based and community-based HIV testing services, an estimated 30% of all people living with HIV are unaware of their HIV-positive status.^1^ Furthermore, since 2010, the number of global new adult HIV infections has remained stable at around 1·9 million per year.^2^ Increasing coverage of HIV prevention services requires novel strategies to deliver HIV testing services and reach individuals who remain unaware of their HIV status.^3^ In Zambia, coverage of HIV testing services has increased substantially since 2007,^4^, ^5^, ^6^ yet there remain gaps. In 2013–14, 46% of women were tested and received the test result within the previous 12 months compared with 37% of men.^6^ During 2015–16, 70% of HIV-positive women knew their HIV-positive status compared with 63% of men.^5^, ^6^ Furthermore, adults older than 24 years are more likely to have tested than adolescents and young people aged 15–24 years.^6^, ^7^

**Research in context**

**Evidence before this study**

We searched PubMed and Medline for English-language publications on studies of strategies to increase HIV testing uptake through distribution of HIV self-tests published through to Sept 14, 2017. We used the search terms self-test* AND HIV infections AND Africa. Of the studies identified, many explored the acceptability and accuracy of HIV self-testing in Kenya, Malawi, and South Africa. These studies consistently reported that HIV self-testing is acceptable, and in Malawi is the preferred option for future HIV testing. Studies exploring the distribution of HIV self-tests included two studies to promote male partner HIV testing: a cohort study of secondary distribution by HIV-negative female sex workers and women receiving antenatal care in Kenya, and a trial of secondary distribution by women receiving HIV self-tests through antenatal care or postpartum care in Kenya. In the cohort study, a large proportion of the women receiving an HIV self-test distributed these to their sexual partners. In the trial, partner HIV testing was higher in the HIV self-test group than in the group offered an invitation for male partners to attend clinic-based HIV testing. In Malawi, a community-based study of HIV self-test distribution showed high uptake and acceptability of HIV self-tests delivered by volunteer counsellors. We found little evidence of rigorous, randomised, controlled trials of strategies to distribute HIV self-tests. Further, there was little available evidence of secondary distribution of HIV self-tests outside of facility settings.

**Added value of this study**

This cluster randomised trial provided rigorous evidence that a 3-month intervention of door-to-door distribution of HIV self-tests increased knowledge of HIV status among adults aged 16 years and older. This effect was driven by increased knowledge of HIV status among men and young adults aged 16–29 years, with no between-group differences noted in women. This was the first trial to show that community-based door-to-door secondary distribution of HIV tests can increase HIV testing among individuals, primarily men, who are not at home during household visits from lay counsellors. Among men aged 30 years or more who HIV self-tested, more than a third self-tested through unsupervised HIV self-testing or secondary distribution of HIV self-tests. The trial also showed that the door-to-door distribution of HIV self-tests increased knowledge of HIV status among community residents whose HIV status was not known to lay counsellors despite 2 years of household delivery of HIV-related services, including HIV testing.

**Implications of all the available evidence**

Our findings suggest that the door-to-door distribution of HIV self-tests increases knowledge of HIV status among individuals who are underserved by currently available HIV testing services. Secondary distribution of HIV self-tests through this strategy was acceptable and might dentify a higher proportion of individuals testing HIV positive. Future research should explore targeted secondary distribution of HIV self-tests to partners of HIV-positive individuals to support reaching individuals at highest risk of infection. The door-to-door distribution of HIV self-tests is a promising strategy that complements currently available HIV testing strategies by accessing so-called harder to reach individuals, including men. Door-to-door secondary distribution of HIV self-tests in high-prevalence settings could support reaching older men, who are more likely to be HIV positive than younger men, and linking them to prevention or treatment services, and reach HIV Prevention 2020 targets.

HPTN 071 (PopART) is a cluster-randomised trial ongoing in 21 communities in South Africa and Zambia to estimate the effect of universal HIV testing and immediate treatment on HIV incidence.^8^ After one round of door-to-door delivery of HIV testing services in the PopART intervention communities in Zambia, the first 90 of the UNAIDS 90-90-90 targets was nearly reached among women, but among men coverage was approximately 10–15% below target.^9^ PopART has shown that, even with intensive household delivery of HIV testing and related services, challenges remain in reaching the ambitious first and second 90s of the UNAIDS 90-90-90 targets, particularly among men.^9^

HIV self-testing is a novel strategy that has the potential to reach individuals underserved by currently available HIV testing strategies.^10^ A systematic review^11^ showed that HIV self-testing is feasible for populations in high HIV-prevalence settings. Since 2016, WHO has recommended evidence-based approaches to delivering HIV self-testing services to reach men and other key populations.^12^ Evidence from Malawi and Kenya suggests that oral HIV self-testing is acceptable and accurate, and can potentially increase community levels of HIV testing and promote male partner testing.^11^, ^13^ Although feasible and acceptable, there remains a need for evidence of how to deliver HIV self-testing services to increase knowledge of HIV status, particularly among men and younger adults.

Here, we report results from a cluster-randomised trial of HIV self-testing services nested within the HPTN 071 (PopART) trial. The nested trial offered an opportunity to evaluate whether the door-to-door offer of the option of oral HIV self-testing alongside the offer of home-based finger-prick rapid diagnostic testing (finger-prick RDT) by lay counsellors increased current knowledge of HIV status among the general adult and adolescent population in four urban communities in Zambia.

## Methods

### Study design and participants

**Figure 1:**
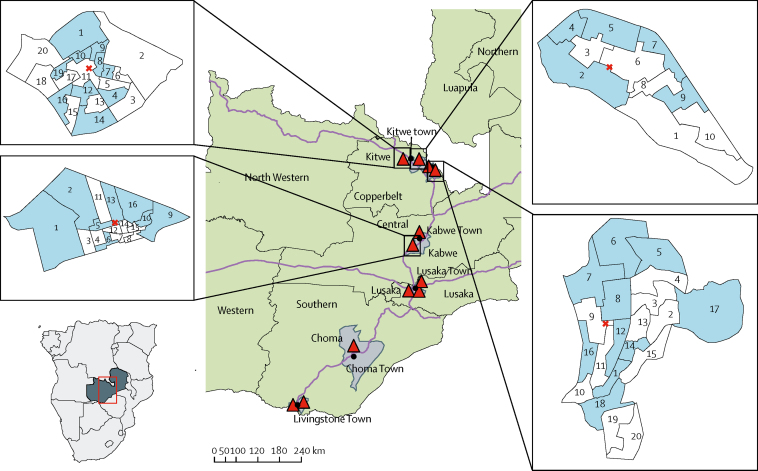
Map showing the randomised zones and location of 12 PopART clusters in Zambia, 2013 Blue areas are zones randomly allocated to the HIV self-testing intervention. Red cross indicates location of the health facility within the community. Black closed circles are towns. Red triangles are HPTN 071 (PopART) communities. Grey areas are PopART districts. Green areas are provinces.

We nested this HIV self-testing cluster-randomised trial in four urban communities in two northern provinces of Zambia (figure 1).^8^ Details of the HPTN 071 (PopART) trial are reported elsewhere.^8^ Briefly, this is a cluster-randomised trial done in 21 communities in Zambia and South Africa to estimate the effect of a household combination HIV prevention package (PopART intervention), which includes the door-to-door offer of HIV testing services (finger-prick RDT), immediate treatment for HIV-positive individuals regardless of CD4 cell count, and promotion of male circumcision for HIV-negative men, on HIV incidence.^8^ Community HIV care providers did annual rounds within a defined geographical area (called a zone), during which they attempt to visit all households, enumerate all household members, and offer the PopART intervention services to all individuals, regardless of previous participation in previous rounds. Throughout an annual round, community HIV care providers return to households to offer HIV testing services to individuals absent at the first household visit, and support linkage to and retention in care for individuals testing HIV-positive.^8^ At the time of the HIV self-testing study, the community HIV care providers were doing their third PopART annual round.^8^

During household visits, data on household enumeration, consent to participate in PopART, and uptake of HIV testing services and other PopART services are collected by community HIV care providers using electronic data capture devices. The four HIV self-testing trial communities were PopART intervention communities selected by the study team to reflect the range and pattern of uptake of HIV testing services in the other PopART intervention communities. Each community had one public health facility, situated a maximum of 1·93–3·67 km from households. Each community had been divided into zones (clusters) that comprised approximately 450–500 households, with an estimated average population of approximately 1400 individuals aged at least 16 years per zone. There were 66 zones across the communities, with an estimated total population of around 90 000 individuals aged at least 16 years. A pair of community HIV care providers managed each zone.

All adolescents and adults aged 16 years and older and resident in the 66 zones were eligible to participate in the HIV self-testing study. During the HIV self-testing study implementation period of approximately 3 months, individuals at home during community HIV care providers' household visits were asked for verbal consent to participate in PopART. If the individual consented and did not report an HIV-positive status, they were eligible for an offer of HIV testing services.

The study was approved by the University of Zambia Biomedical Research Ethics Committee and London School of Hygiene and Tropical Medicine ethics committee. Permission to do the study was also granted by the Division of AIDS at the National Institutes of Health (MD, USA), the Zambia National Health Research Authority, and the Zambia Medicines Regulatory Authority.

### Randomisation and masking

The 66 zones were randomly assigned (1:1) to either oral HIV self-testing plus the PopART standard of care of routine door-to-door HIV testing services (HIV self-testing group) or door-to-door HIV testing services alone (non-HIV self-testing group) over a 3-month period. To achieve balance across clusters in factors that were likely to affect the primary outcome, we stratified randomisation by community.^14^ We also restricted the randomisation, first within each community and second across all four communities, to achieve balance by trial group on average values of key outcomes measured during round 2, including: the percentage of adults whose HIV status was known to the community HIV care providers by the end of round 2; the percentage accepting the offer of HIV-testing among those eligible to test in round 2, overall and separately for men, women, adults aged 18–29 years, and those resident in rounds 1 and 2; the percentage of men not contacted during round 2, and the average number of adults per zone. SF generated the randomisation protocol and, from several billion possible allocations that met restriction criteria, drew a computer-generated random sample from combinations of 10 000 possible allocations.

In December, 2016, we held a randomisation ceremony with community HIV care providers, their supervisors, and members of the PopART community advisory boards to allocate the zones to the HIV self-testing or non-HIV self-testing groups. Using the randomly selected list of allocations numbered from 0000 to 9999, four individuals selected four numbered balls from a bag. This four-digit number corresponded to an allocation number in the list of 10 000, and determined, for each zone, whether it was to be allocated to group 0 or 1. In a second stage, a similar process was used to randomly assign the zones numbered 0 and 1 to either the HIV self-testing or non-HIV self-testing groups (figure 1). As a cluster-randomised trial of a strategy to deliver HIV self-testing services to all households within a cluster, blinding of participants and community HIV care providers was not feasible.

### Procedures

The intervention was implemented between 18 Jan, and 30 April, 2017. The first 2 weeks of implementation were considered a priori pre-full-implementation weeks. As such, the evaluation period was from Feb 1, to April 30, 2017. In zones randomised to the HIV self-testing intervention, individuals contacted during community HIV care providers household visits who consented to participate in PopART and were eligible for HIV testing were offered a choice of using oral HIV self-testing or a finger-prick sampling of whole blood and rapid HIV testing (finger-prick RDT). This was done according to PopART procedures and the Zambian national HIV testing algorithm, using Alere Determine HIV-1/2 (Chiba, Japan) as the screening test and Uni-Gold HIV test (ref 1206502, Trinity Biotech, Ireland) as the confirmatory test. For individuals choosing HIV self-testing, community HIV care providers demonstrated how to do the self-test and read the result. Because HIV self-testing was a novel procedure in the four communities, individuals were offered either supervised (in the presence of a community HIV care provider) or unsupervised (in the absence of a community HIV care provider) HIV self-testing. The level of supervision and support provided was dependent on individual preference. Individuals opting for HIV self-testing were given a package consisting of the OraQuick self-test kit (Bangkok, Thailand),^15^ user-friendly pictorial instructions for use provided by the manufacturer (translated into local languages), a card with the community HIV care provider's phone number, a self-complete results form, and an envelope for returning the used self-test.

Individuals aged 18 years or older with a partner living in the same household but absent at the time of the community HIV care provider's visit were offered an HIV self-test kit for their absent partner. Individuals accepting an HIV self-test kit for secondary distribution were asked to sign an agreement stating that the kit would only be given to the intended individual, that the individual would not be coerced into using the HIV self-test kit, and that the information required for the individual to use the HIV self-test kit would be communicated to them. The community HIV care provider left a card with their phone number to allow the absent individual to contact them should additional support be required and for linkage to services, including confirmatory testing.

For individuals choosing unsupervised HIV self-testing or where an HIV self-test kit was left for an absent partner, community HIV care providers did a follow-up visit within 7 days to verify use and offer follow-up services. Individuals also had the option of returning the HIV self-test package to the clinic. During follow-up visits, community HIV care providers collected HIV self-test kits and self-completed results forms where available. The community HIV care providers also attempted to meet individuals who received an HIV self-test kit via their partner. Individuals whose HIV self-test results were recorded in their absence were considered to have participated in the PopART intervention in round 3. Where the HIV self-test kit was available, the community HIV care providers checked the results of the test, and for individuals who they met in person and whose HIV self-test result was reactive, they offered confirmatory HIV testing using parallel testing with the same rapid diagnostic tests as HIV finger-prick RDT. Post-test counselling was provided, and HIV-positive individuals were referred to treatment and care irrespective of whether they accepted the offer of confirmatory testing. HIV-negative individuals were counselled and referred to HIV prevention services. Individuals who were left an HIV self-test kit during the intervention period were followed up until Sept 30, 2017 to provide support on doing the HIV self-testing, confirmatory testing, and linking to services as required.

Zones randomised to the non-HIV self-testing group continued to receive the standard PopART combination package of interventions, including the offer of home-based HIV finger-prick RDT. For individuals testing HIV positive, community HIV care providers provided post-test counselling, and referral to HIV treatment and care services. HIV-negative individuals were counselled and referred to HIV prevention services.

### Outcomes

The primary outcome was current knowledge of HIV status, defined as an individual self-reporting knowing their HIV-positive status to a community HIV care provider, or accepting an offer of either HIV self-testing or finger-prick RDT and the HIV test result was recorded by community HIV care providers. Where an HIV self-test kit was distributed for secondary use, the test was recorded as used if the index individual reported the result to the community HIV care provider or the intended user of the HIV self-test kit later met the community HIV care provider and reported that they had used the test. We measured the outcome among households that were first visited in round 3 between Feb 1, and April 30, 2017, restricted to individual household members aged 16 years or older who were first enumerated or (re-)enumerated as a household member during this period. Information on outcomes used data collected from household visits, and re-visits (where an HIV self-test kit was left for unsupervised or a secondary distribution), done between Feb 1, and June 30, 2017. Individuals aged 16 years or older enumerated as a household member but not seen by the community HIV care provider during the study period were assumed not to know their HIV status in round 3.

Secondary outcomes were consent to participate in PopART; uptake of HIV testing services among individuals who consented to PopART and were eligible for an offer of HIV-testing services; linkage to confirmatory testing in the HIV self-testing group; programmatic costs and incremental cost-effectiveness of adding HIV self-testing to the PopART intervention; and qualitatively describing HIV self-test kit distribution and social harms. Social harms were identified during observations of HIV self-test kit distribution and through community engagement mechanisms, such as stakeholder and community advisory board meetings, and were categorised between incidents related or unrelated to the study and between serious and non-serious incidents. In this Article, we describe only social harms reported during the study period. Further analysis on secondary distribution and linkage to confirmatory testing is ongoing and will be reported elsewhere.

We did sensitivity analyses of the primary outcome to include individuals whose HIV-positive status was known to the community HIV care provider in round 1 or round 2 (or both) of PopART, who were enumerated during the 3-month implementation phase of this trial, but who did not participate, either because they were absent or they did not consent to PopART.

### Statistical analysis

The study was powered to show an overall reduction of 5% in the percentage of adults who did not know their HIV status in the HIV self-testing group compared with the non-HIV self-testing group, assuming that the percentage who did not know their HIV status in the non-HIV self-testing group was in the range 35–40%. Study power was greater than 90% if the between-zone coefficient of variation k was 0·15, and around 70–80% if k was 0·20, assuming an average of approximately 400 adults enumerated per zone. For subgroup analyses by sex, study power was in the range of around 60–90% to show a 5% reduction in the percentage of individuals who did not know their HIV status in the HIV self-testing group, assuming that the percentage who did not know their HIV status in the non-HIV self-testing group was in the range 30–45% and that k was 0·2.

To estimate the effect of the HIV self-testing intervention, we analysed data at the individual level, using population-average logistic regression models to account for clustering by zone, to adjust for community to explain some of the between-zone variation, and a priori for age group and sex. Prespecified sub-group analyses for the primary and secondary outcomes included analyses by sex, age (individuals aged 16–29 years, adults aged ≥30 years), and individuals who were resident during rounds 1 and 2 of PopART but who did not participate in either round. We analysed the data with Stata version 15.0.

We did a prospective economic evaluation, from the provider's perspective, to comparatively calculate unit costs of HIV testing services in both groups and calculated the incremental cost of delivering HIV testing services in the HIV self-testing group. Full annual financial and economic costs were calculated. Financial costs included all expenditures for resources in both groups, whereas economic costs captured the full value of all resources used to deliver HIV testing services in both groups, including the valuation of donated goods or services and individual time to deliver services.^16^ Cost inputs included equipment, HIV testing supplies, general supplies, transportation and travel, administration, and personnel resources (appendix p 3). In this analysis, we used landed costs of US$3·00 per HIV self-test kit, which accounted for purchase, shipment, and landing taxes. Resource use data were collected between Dec 1, 2016, and June 30, 2017. Costs were adjusted to 2017 US$ using an assumed exchange rate of ZMW 9·50.

Data sources included financial records, the community HIV care provider's electronic data capture device, and interviews with the HIV self-testing intervention team. In the cost analysis, we calculated the total cost of implementing HIV testing service activities, and cost per person who was: enumerated, tested, and newly diagnosed HIV positive for both groups.

### Role of the funding source

The International Initiative for Impact Evaluation (3ie) reviewed and provided non-binding comments on the draft manuscript before submission. The other funders of the study had no role in study design, data collection, data analysis, data interpretation, or writing of the report. All authors had final responsibility for the decision to submit for publication.

## Results

**Figure 2:**
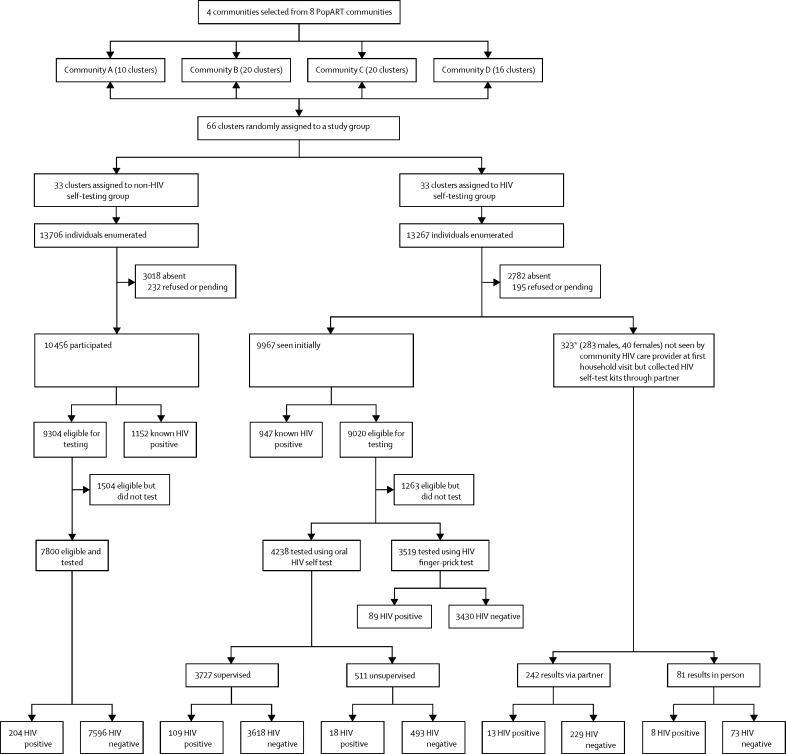
Enumeration and uptake of HIV testing in the HIV self-test and non-HIV self-test groups Pending refers to individuals who made appointments but were not yet seen by a community HIV care provider as of June 30, 2017. *3/323 individuals were identified as knowing their HIV positive status before self-testing in the absence of the community HIV care provider after they were subsequently contacted in PopART annual round 3.

Between Feb 1, and April 30, 2017, the community HIV care providers enumerated 13 267 eligible individuals in the HIV self-testing group and 13 706 in the non-HIV self-testing group (table 1, figure 2). In both groups, half the individuals were aged 16–29 years, and a similar proportion were absent during the community HIV care provider's household visit (table 1).

**Table 1:** Baseline characteristics of the study population

		**HIV self-testing (n=13 267)**	**Non-HIV self-testing (n=13 706)**
Male sex		6368 (48%)	6486 (47%)
Age group (years)			
	16–19	2176 (16%)	2190 (16%)
	20–24	2653 (20%)	2804 (21%)
	25–29	1940 (15%)	2008 (15%)
	30–34	1651 (12%)	1641 (12%)
	35–44	2355 (18%)	2345 (17%)
	≥45	2492 (19%)	2718 (20%)
Absent during community HIV care-provider's visit			
	Total	2782 (21%)	3018 (22%)
	Men	1942 (70%)	2140 (71%)
	Women	840 (30%)	878 (29%)
Self-reported HIV positive (percentage of those present)		950 (9%)	1152 (11%)
Eligible for HIV testing		9340 (91%)	9304 (89%)
Previously participated in PopART (in same community HIV care-provider zone)		8093 (61%)	8745 (64%)
Previously resident in PopART annual rounds 1 or 2 (in same community HIV care-provider zone)		9376 (71%)	9946 (73%)

Data are n (%). The HIV self-testing group received oral HIV self-testing plus routine door-to-door HIV testing. The non-HIV self-testing group received only routine door-to-door HIV testing.

After the intervention period, 9027 (68%) of 13 267 in the HIV self-testing group knew their HIV status compared with 8952 (65%) of 13 706 individuals in the non-HIV self-testing group (adjusted odds ratio [OR] 1·30, 95% CI 1·03–1·65; p=0·03; table 2).

**Table 2:** Knowledge of HIV status

	**HIV self-testing**	**Non-HIV self-testing**	Adjusted odds ratio*(95% CI)	**p value**
Overall	9027/13267 (68%)	8952/13706 (65%)	1·30 (1·03–1·65)	0·03
Men	3843/6368 (60%)	3571/6486 (55%)	1·31 (1·07–1·60)	0·01
Women	5184/6899 (75%)	5381/7220 (75%)	1·05 (0·86–1·30)	0·62
Young adults (age 16–29 years)	4972/6769 (74%)	4917/7002 (70%)	1·31 (1·05–1·63)	0·02
Older adults (≥30 years)	4055/6498 (62%)	4035/6704 (60%)	1·22 (0·98–1·52)	0·07
Resident in PopART annual rounds 1 and 2, but did not participate in either round	173/583 (30%)	117/567 (21%)	1·63 (1·15–2·31)	0·01
Participated in PopART annual rounds 1 or 2 or both, but declined door-to-door HIV testing	600/1344 (45%)	587/1425 (41%)	1·29 (0·95–1·76)	0·11

Data are n/N (%). The HIV self-testing group received oral HIV self-testing plus routine door-to-door HIV testing. The non-HIV self-testing group received only routine door-to-door HIV testing.

* Adjusted for sex, age, community, and clustering by zones.

There was evidence that the effect of the intervention differed by sex (p_interaction_=0·01). Among men, knowledge of HIV status was higher in the HIV self-testing group than in the non-HIV self-testing group whereas among women, knowledge of HIV status did not differ between groups (table 2). The effect did not seem to differ by age group (p_interaction_=0·44), but more adults aged 16–29 years and 30 years or older in the HIV self-testing group knew their HIV status compared with the non-HIV self-testing group (table 2).

Similarly, among individuals who were resident during PopART rounds 1 and 2, but did not participate in either round, a greater number in the HIV self-testing group had knowledge of their HIV status compared with the non-HIV self-testing group (table 2).

Stratified by age and sex, knowledge of HIV status was higher in the HIV self-testing group than in the non-HIV self-testing group among younger and older men, men who were resident during PopART rounds 1 and 2 but who did not participate in either round, and men who participated in previous rounds of PopART but declined HIV testing (table 3). There was weak evidence that the intervention had an effect among the small number of women resident in PopART rounds 1 and 2 but who did not participate in either round, with little evidence of an effect among women who participated in previous rounds of PopART but declined HIV testing (table 3).

**Table 3:** Knowledge of HIV status stratified by sex

	**Men**				**Women**			
	HIV self-testing	Non-HIV self-testing	Adjusted odds ratio* (95% CI)	p value	HIV self-testing	Non-HIV self-testing	Adjusted odds ratio* (95% CI)	p value
Overall	3843/6368 (60%)	3571/6486 (55%)	1·31 (1·07–1·60)	0·01	5184/6899 (75%)	5381/7220 (75%)	1·05 (0·86–1·30)	0·62
Young adults (age 16–29 years)	2091/3129 (67%)	1979/3233 (61%)	1·31 (1·04–1·65)	0·02	2881/3640 (79%)	2938/3769 (78%)	1·15 (0·93–1·44)	0·21
Older adults (age ≥30 years)	1752/3239 (54%)	1592/3253 (49%)	1·37 (1·10–1·72)	0·01	2303/3259 (71%)	2443/3451 (71%)	1·01 (0·80–1·27)	0·96
Resident in PopART annual rounds 1 and 2, but did not participate in either round	128/441 (29%)	86/427 (20%)	1·64 (1·15–2·35)	0·01	45/142 (32%)	31/140 (22%)	1·68 (1·02–2·77)	0·04
Participated in PopART annual rounds 1 or 2 or both, but declined door-to-door HIV testing	282/656 (43%)	236/665 (36%)	1·47 (1·03–2·09)	0·03	318/688 (46%)	351/760 (46%)	1·05 (0·78–1·41)	0·73

Data are n/N (%). The HIV self-testing group received oral HIV self-testing plus routine door-to-door HIV testing. The non-HIV self-testing group received only routine door-to-door HIV testing.

* Adjusted for sex, age, community, and clustering by zones.

In a sensitivity analysis, including individuals whose HIV-positive status was known to community HIV care providers in PopART rounds 1 or 2, or both, but who had not participated in this HIV self-testing trial, 9179 (69%) of 13 267 knew their HIV status in the HIV self-testing group compared with 9079 (66%) of 13 706 in the non-HIV self-testing group (adjusted OR 1·34, 95% CI 1·07–1·69; p=0·01). Among men, 3910 (61%) of 6368 knew their HIV status in the HIV self-testing group compared with 3622 (56%) of 6486 in the non-HIV self-testing group (adjusted OR 1·34, 95% CI 1·10–1.64, p=0·004). After accounting for variation explained by differences among the four communities and the effect of the HIV self-testing intervention, the between-zone coefficient of variation (k) for the percentage of individuals who did not know their HIV status by the end of this trial was 0·23.

Participation in PopART was slightly higher in the HIV self-testing group, with 10 290 (78%) of 13 267 enumerated, either because they were contacted and consented to participate, or because they self-tested through secondary distribution and their results were reported to and recorded by the community HIV care providers. In the non-HIV self-testing group, 10 456 (76%) of 13 706 enumerated individuals participated in PopART (adjusted OR 1·40, 95% CI 0·98–1·99; p=0·06). Stratified by sex, more men in the HIV self-testing group than in the non-HIV self-testing group participated in PopART, but there were no between-group differences in women (table 4). By sex and age, there was little evidence of an effect among women of either age group, or among men aged 16–29 years.

**Table 4:** Participation in the PopART intervention and accepting an offer of HIV testing

	**Men**				**Women**			
	HIV self-testing	Non-HIV self-testing	Adjusted odds ratio (95% CI)*	p value	HIV self-testing	Non-HIV self-testing	Adjusted odds ratio (95% CI)*	p value
**Participation in the PopART intervention**								
Overall	4331/6368 (68%)	4219/6486 (65%)	1·27 (0·99–1·63)	0·06	5959/6899 (86%)	6237/7220 (86%)	1·00 (0·77–1·30)	0·99
Young adults (age 16–29 years)	2281/3129 (73%)	2273/3233 (70%)	1·18 (0·91–1·52)	0·21	3176/3640 (87%)	3297/3769 (88%)	1·04 (0·82–1·33)	0·74
Older adults (age ≥30 years)	2050/3239 (63%)	1946/3253 (60%)	1·29 (0·98–1·69)	0·07	2783/3259 (85%)	2940/3451 (85%)	0·97 (0·71–1·33)	0·86
Resident in PopART annual rounds 1 and 2, but did not participate in either round	148/441 (34%)	105/427 (25%)	1·59 (1·08–2·33)	0·02	64/142 (45%)	54/140 (39%)	1·20 (0·71–2·03)	0·50
Participated in PopART annual rounds 1 or 2 or both, but declined door-to-door HIV testing	402/656 (61%)	390/665 (59%)	1·26 (0·85–1·87)	0·24	547/688 (80%)	604/760 (80%)	0·99 (0·66–1·48)	0·95
**Accepting an offer of HIV testing services among individuals eligible for HIV testing**								
Overall†	3581/4069 (88%)	3242/3890 (83%)	1·42 (1·10–1·85)	0·01	4496/5271 (85%)	4558/5414 (84%)	1·05 (0·82–1·35)	0·68
Young adults (age 16–29 years)	2063/2253 (92%)	1945/2239 (87%)	1·56 (1·15–2·12)	0·01	2714/3009 (90%)	2731/3090 (88%)	1·29 (0·95–1·74)	0·10
Older adults (age ≥30 years)	1518/1816 (84%)	1297/1651 (79%)	1·44 (1·07–1·94)	0·02	1782/2262 (79%)	1827/2324 (79%)	1·02 (0·79–1·33)	0·85
Resident in PopART annual rounds 1 and 2, but did not participate in either round	122/142 (86%)	83/102 (81%)	1·33 (0·66–2·66)	0·42	43/62 (69%)	26/49 (53%)	1·74 (0·76–3·98)	0·19
Participated in PopART annual rounds 1 or 2 or both, but declined door-to-door HIV testing	276/396 (70%)	223/377 (59%)	1·64 (1·05–2·54)	0·03	300/529 (57%)	327/580 (56%)	1·02 (0·71–1·45)	0·93

Data are n/N (%).

* Adjusted for sex, age, community, and clustering by zones.

† Three individuals who first used an HIV self-test kit in the absence of a community HIV care provider but later self-reported being HIV positive were not included.

The intervention increased acceptance of an offer of HIV testing services among men but not women (table 4, appendix pp 5, 6). The effect was similar among young men aged 16–29 years and men aged 30 years and older. There were no between-group differences in accepting HIV testing services in either men or women who were previously resident in PopART rounds 1 and 2 but did not participate in either round. Among men who participated in previous rounds of PopART but declined HIV testing, HIV testing uptake was greater in the HIV self-testing group (table 4). There was no evidence of an effect among women (table 4).

Among individuals who opted for HIV self-testing in the HIV self-testing group, most chose supervised HIV self-testing, with the number of supervised HIV self-tests higher in women than men (appendix p 2). Among women, the method of HIV self-testing differed little by age group (appendix p 2). Among men, the type of HIV self-testing differed by age: 325 (38%) of 847 men aged 30 years and older who self-tested used unsupervised or secondary distribution HIV self-test kits, compared with 199 (17%) of 1161 men aged 16–29 years (p=0·004). Among the 148 individuals whose first HIV self-testing result was reactive (figure 2), seven (5%) subsequently reported they had known they were HIV positive before self-testing, and three (2%) who had tested via secondary distribution subsequently did a repeat HIV self-testing after meeting the community HIV care providers and the test result was negative. Of the remaining 138 who were eligible for confirmatory HIV testing, 105 (76%) linked to confirmatory testing, of whom 102 (97%) were confirmed HIV positive.

The total cost of delivering HIV testing services was US$243 745 in the HIV self-testing group and US$172 069 in the non-HIV self-testing group. Personnel costs formed the largest proportion of the total costs in both groups followed by testing supplies, with the remaining costs being resource inputs at less than 10% in each group (appendix p 4). HIV self-testing activities accounted for $84 135 (35%) of the $243 745 cost of implementing HIV testing services in the HIV self-testing group.

The cost per person tested in the HIV self-testing group was 1·37 times higher than in the non-HIV self-testing group. The incremental costs of distributing HIV self-test kits door-to-door alongside routine door-to-door HIV testing services was estimated at $71 675·78, which resulted in an incremental cost per additional person tested of $255·98 ($71 675·78 total costs for 280 individuals). Incremental costs per individual confirmed HIV self-test-positive was calculated at $771·88 ($84 135 total costs for 109 individuals; appendix p 4).

13 social harms occurred in the HIV self-testing group. These ranged from invasion of privacy, emotional distress, being deceived or forced into doing HIV testing, threatening violence, to actual violence, and separation of couples (appendix p 7). Some social harms were exacerbated by pre-existing conditions within a couple, such as alcohol abuse and a history of gender-based violence.

## Discussion

Our findings showed that a 3-month intervention of a door-to-door offer of HIV self-testing as an option for HIV testing had small but significant effects on current knowledge of HIV status among the general population aged 16 years or older in four communities in Zambia. The intervention had an overall effect among men, but not women, and an effect among men and women resident in the communities during rounds 1 and 2 of PopART but who did not participate in either round. Participation in PopART increased among men but not women, with an increase in HIV testing among men who previously declined HIV testing services and a small increase among women who previously declined participation in PopART.

In Zambia, like other southern African countries, men are harder to reach with HIV testing services.^17^ HIV self-testing has been proposed as a strategy to reach men.^18^, ^19^ We found that the door-to-door offer of the HIV self-test option increased men's knowledge of HIV status and uptake of HIV testing services. This effect was driven by increased acceptability of HIV self-testing compared with standard finger-prick RDT, and by secondary distribution of HIV self-testing kits. Almost all individuals reached through secondary distribution were male, and almost half the men aged 30 years and older who self-tested did so through unsupervised or secondary distribution of HIV self-testing kits. This study is among the first to evaluate community-based secondary distribution of HIV self-test kits. The available evidence shows that secondary distribution is feasible and effective when offered to antenatal care attendees, postpartum women, or female sex workers.^11^, ^20^, ^21^ A Kenyan trial^22^ comparing secondary distribution of HIV self-test kits with an invitation for partners of antenatal care attendees and postpartum women to attend facility-based HIV-testing services showed that secondary distribution increased partner testing by 39%. Our study adds to the available evidence suggesting that community-based secondary distribution is effective at reaching men in communities exposed to 3 years of door-to-door delivery of HIV testing services.

Similar to findings from a Malawian HIV study,^13^ in which 25% of individuals self-testing after 2 years of service promotion were aged younger than 20 years, we showed that the HIV self-testing intervention had small but significant effects on knowledge of HIV status and uptake of HIV testing services among younger individuals aged 16–29 years.^23^, ^24^ HIV incidence is high among adolescents and young people, particularly among females aged 15–24 years who are at highest risk of HIV in sub-Saharan Africa.^2^ Adolescents and young people, particularly younger men, are harder to reach with HIV services, and less likely to be engaged in all steps of the HIV care cascade.^24^ In PopART, door-to-door HIV testing services increased HIV testing uptake and knowledge of HIV status among adolescents aged 15–19 years; however, 28% of adolescents were not reached, mainly because men and younger age groups were absent during household visits.^25^ HIV self-testing provides a crucial opportunity to reach this underserved population.

Our findings suggest that HIV self-testing reached previously unreached individuals, including individuals previously resident in the community but who did not participate in two rounds of PopART.^9^, ^26^ Most individuals who self-tested opted for supervised HIV self-testing. HIV self-testing probably displaced use of finger-prick RDT by some individuals who would have otherwise accepted finger-prick HIV-testing services, but the use of HIV self-testing was new in these communities and testing preferences would probably change over time with increased familiarity. The OraQuick HIV self-test used in this study has a sensitivity of 95·5% (95% CI 89·7–98·5) when compared with the Zambian national rapid diagnostic test algorithm and is more expensive than standard blood-based rapid diagnostic tests.^15^ Our cost analysis showed that the economic cost per HIV tester was higher in the HIV self-testing group than in the non-HIV self-testing group. As lay counsellors become more familiar with offering HIV self-testing and communities more aware of HIV self-testing, unit costs are likely to decrease with time. Costs of accessing harder-to-reach individuals might, however, be higher still in settings where there has been little access to HIV testing services. In this study setting, many HIV-positive individuals were reached after 3 years of PopART service delivery, and therefore HIV positivity in testers was lower than in a population naive to HIV testing.

The HIV positivity of supervised HIV self-testing, unsupervised HIV self-testing, and finger-prick RDT were similar. Through secondary distribution, HIV positivity was slightly higher than primary HIV self-test kit distribution and finger-prick RDT. Although the numbers were small, these findings suggest that offering HIV self-testing to individuals who are home during household visits reaches a similar population as finger-prick RDT. Secondary distribution and unsupervised HIV self-testing among men, however, seems to reach individuals that would have been missed had only finger-prick RDT been available. However, confirming use of an HIV self-test kit, and measuring linkage to care is challenging with unsupervised HIV self-testing and secondary distribution. In a Kenyan trial of truck drivers, there was no difference in HIV testing uptake comparing the choice of finger-prick RDT or supervised HIV self-testing with an offer of finger-prick RDT.^27^ However, when including men who took an HIV self-testing kit home and self-reported use via the telephone, there was evidence that the choice group had higher testing uptake.^27^ In our study, among individuals who used a secondary distribution HIV self-test kit and for whom community HIV care providers recorded a reactive result, more than a third were later seen by the community HIV care provider and confirmed HIV positive. For many, HIV self-testing is probably appealing as it is private and confidential. This benefit, however, poses challenges for public health research to measure the effect of HIV self-testing on the uptake of HIV testing services, linkage to care, or prevention services. Novel strategies to measure uptake and linkage, or non-financial incentives to encourage return of used HIV self-test kits, could be explored in future studies.

The expansion of HIV self-testing has been met with concerns regarding potential for social harm.^11^, ^28^ A review of studies of HIV self-testing found little published evidence of social harms associated with HIV self-testing.^28^ We noted little evidence of serious adverse events attributable to HIV self-testing in our study. We consider this to be because community HIV care providers were careful about how they introduced HIV self-testing and, in the case of secondary distribution, informed individuals to be cautious when introducing HIV self-test kits to partners. Further, HIV self-test kits were only left for absent partners of individuals aged 18 years and older. This could, however, also be partly due to a reluctance of community members to discuss negative social experiences with researchers. The occurrence and reporting of less severe social harms, such as coerced HIV testing, suggests that developing mechanisms for detecting and reporting social harms earlier in a study might be beneficial.

Our study had limitations. The HIV self-testing intervention was done in established PopART communities where trained community HIV care providers have built good rapport with the community and have been providing HIV testing services since 2013. This exposure to door-to-door testing services might have affected uptake of HIV self-testing and might limit the generalisability of the findings. It is also likely that providing HIV self-test kits in this setting increased the costs because a more pragmatic approach would be taken in a real-life setting. By its nature, an HIV self-test kit is meant to be used in private. As such, the results of use of secondary distribution HIV self-test kits might have inherent biases. However, we believe reporting bias was minimised given the established relationships between the community HIV care providers and the community. Although blinding of the participants was not feasible, performance bias was minimised by allocating the community HIV care providers permanently to either the HIV self-testing group or the non-HIV self-testing group throughout the study.

In conclusion, a 3-month intervention of the addition of HIV self-testing to door-to-door offer of finger-prick RDT had small but significant effects on knowledge of HIV status and uptake of HIV testing services in four communities in Zambia. This effect was seen among men, but also among community residents who previously declined participation in PopART. Community-based secondary distribution of HIV self-test kits might be an effective strategy to provide HIV testing to reach individuals underserved by HIV-testing services in settings exposed to door-to-door delivery of HIV-testing services. To maximise the effect and reduce costs, any future rollout plan should target services more efficiently to reach men and other populations who are not currently accessing available HIV-testing services.
