# Supplementary material for: Community based distribution of oral HIV self-testing kits in Zambia: a cluster-randomised trial nested in four HPTN 071 (PopART) intervention communities
Source: Lancet HIV. 2018 Dec 21;6(2):e81–92. doi: 10.1016/S2352-3018(18)30258-3 (PMC6361868; doi:10.1016/S2352-3018(18)30258-3)
Supplement: Supplementary appendix [file mmc1.pdf]

THE LANCET
HIV

Supplementary appendix

This appendix formed part of the original submission and has been peer reviewed. We post it as supplied by the authors.

Supplement to: Mulubwa C, Hensen B, Phiri MM, et al, for the HPTN 071 (PopART) Study Team. Community based distribution of oral HIV self-testing kits in Zambia: a cluster-randomised trial nested in four HPTN 071 (PopART) intervention communities. *Lancet HIV* 2018; published online Dec 21. http://dx.doi.org/10.1016/S2352-3018(18)30258-3.

Appendices material for "Community based distribution of oral HIV self-testing kits in Zambia: a cluster-randomized trial nested in four HPTN 071 (PopART) intervention communities"

Appendices

1. Type of HIV testing services selected by individuals eligible for an offer of and choosing to test for HIV in HIV self-testing zones
2. Description of cost components
3. Unit costs (US$) by HIV self-testing and non-HIV self-testing groups
4. Enumeration and uptake of HIV testing in the HIV self-testing and non-HIV self-testing groups among males
5. Enumeration and uptake of HIV testing in the HIV self-testing and non-HIV self-testing among females
6. Social harms matrix

Appendix 1. Type of HIV testing services selected by individuals eligible for an offer of and choosing to test for HIV in HIV self-testing zones

	HIV self-testing				HIV finger-prick RDT (%, n/N)	Overall (%, n/N)
	**Supervised (%, n/N)**	**Unsupervised (%, n/N)**	**Secondary distribution (%, n/N)**	**Overall (%, n/N)**		
**Overall**	81.7 (3,727/4,561)	11.2 (511/4,561)	7.1 (323/4,561)	56.4 (4,561/8080)	43.6 (3,519/8,080)	86.5 (8,080/9,343)
**Male**	73.9 (1,484/2,008)	12.0 (241/2,008)	14.1 (283/2,008)	56.1 (2,008/3,582)	43.9 (1,574/3,582)	88.0 (3,582/4,070)
**Female**	87.9 (2,243/2,553)	10.6 (270/2,553)	1.6 (40/2,553)	56.8 (2,553/4,498)	43.2 (1,945/4,498)	85.3 (4,498/5,273)
**Overall 16-29**	85.8 (2,327/2,712)	10.2 (277/2,712)	4.0 (108/2,712)	56.8 (2,712/4,777)	43.2 (2,065/4,777)	90.8 (4,777/5,262)
**Males 16-29**	82.9 (962/1,161)	9.8 (114/1,161)	7.3 (85/1,161)	56.3 (1,161/2,063)	43.7 (902/2,063)	91.6 (2,063/2,253)
**Female 16-29**	88.0 (1,365/1,551)	10.5 (163/1,551)	1.5 (23/1,551)	57.1 (1,551/2,714)	42.9 (1,163/2,714)	90.2 (2,714/3,009)
**Overall 30+**	75.7 (1,400/1,849)	12.7 (234/1,849)	11.6 (215/1,849)	56.0 (1,849/3,303)	44.0 (1,454/3,303)	80.9 (3,303/4,081)
**Male 30+**	61.6 (522/847)	15.0 (127/847)	23.4 (198/847)	55.8 (847/1,519)	44.2 (672/1,519)	81.2 (1,519/1,871)
**Female 30+**	87.6 (878/1,002)	10.7 (107/1,002)	1.7 (17/1,002)	56.2 (1,002/1,784)	43.8 (782/1,784)	78.8 (1,784/2,264)

Appendix 2: Description of cost components

Component	Description
**Intervention costs**	These were the additional costs of introducing HIV self-testing into PopART. Costs of HIV self-testing: procuring HIV self-testing kits, including the actual cost of kits and transportation up to central level. Cost of supplies: including cost of supplies that directly support implementation of the intervention; e.g. stationary (including teaching aid reproduction) and extra bags for the HIV self-testing kits. Personnel costs.
**Research Costs**	Included costs which were related to impact evaluation, social science and economic evaluation
**Community Sensitization and mobilization**	Costs of additional community engagement activities related to HIV self-testing distribution including personnel, supplies, transportation and travel and capital costs. For example the cost of sensitization meetings with Ministry of Health at central, provincial and district levels and community sensitization meetings.
**Quality Assurance**	Costs of specific QA/QC activities related to HIV self-testing disaggregated by personnel, supplies, transportation and travel, and capital costs.
**Project coordination**	These generally included shared costs related to administrative and project coordination activities including supervision and mentorship, mostly incurred at central office. Costs associated with technical support from central office – travel costs such as per diem, accommodation and transportation – including personnel costs.
**Set-up costs**	These are costs which were incurred before 1 February 2017 to set up the project
**Trainings**	Costs of initial trainings; Initial protocol training for field staff.
**PopART Community intervention costs**	All costs related to PopART community finger prick RDT activities
**Cost of the intervention (HIVST) Arm**	Intervention costs plus PopART community intervention costs
**Cost of non-HIV self-testing**	PopART community intervention costs

Appendix 3. Unit costs (US$) by HIV self-testing and non-HIV self-testing groups

Item	Non-HIV self-testing group	HIV self-testing group
Total intervention costs	***Value (US$)***	***Value (US$)***
PopART Costs		
**Staff costs**	145,694	145,694
**General supplies**	3,693	3,693
**First line testing supplies**	22,004	9,927
**Second line test supplies**	677	296
Sub-total costs	*172,069*	*159,610*
HIV self-testing costs		
**Personnel**		48,456
**Administration**		9,844
**Transportation & Travel**		6,796
**Supplies**		4,089
**HIVST kit**		13,683
**Equipment**		613
**Parallel HIV testing***		654
Sub-total costs		*84,135*
**Total Costs**	**172,069**	**243,745**
Outcomes	***number***	***number***
**Number of persons enumerated**	13,706	13,267
**Number of persons tested**	7800	8080
**Number of Newly HIV+ persons identified**	204	237
**HIVST kits distributed**		4561
**Number confirmed HIV self-testing HIV+ persons[&]**		109
**Incremental number of individuals tested**		280
Cost-effectiveness (US$)	***Value (US$)***	***Value (US$)***
**Cost per person enumerated**	12.55	18.37
**Cost per person tested**	22.06	30.17
**Cost per newly HIV+ person identified**	843.47	1028.46
**Cost per HIV self-test kit distributed**		18.45
**Cost per confirmed HIV self-testing HIV+**		771.88
**Incremental costs**		71,675.78
**Incremental cost per person tested**		255.98

Key: [&]Confirmed HIV self-testing HIV+ - individuals who self-tested HIV positive and accepted a confirmatory test
*Parallel HIV testing is when Determine HIV and UniGold tests are performed to confirm an HIV test result

Appendix 4: Enumeration and uptake of HIV testing in the HIV self-testing and non-HIV self-testing groups among males

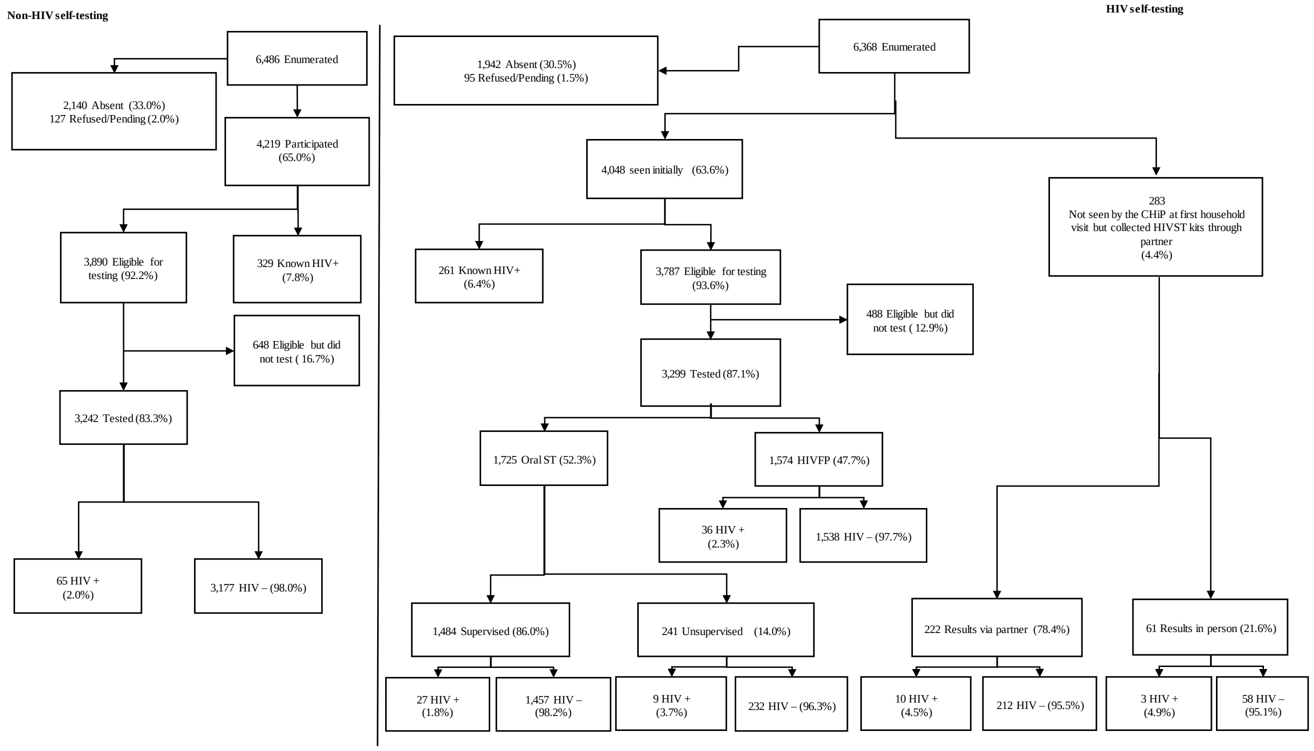

Appendix 5: Enumeration and uptake of HIV testing in the HIV self-testing and non-HIV self-testing groups among females

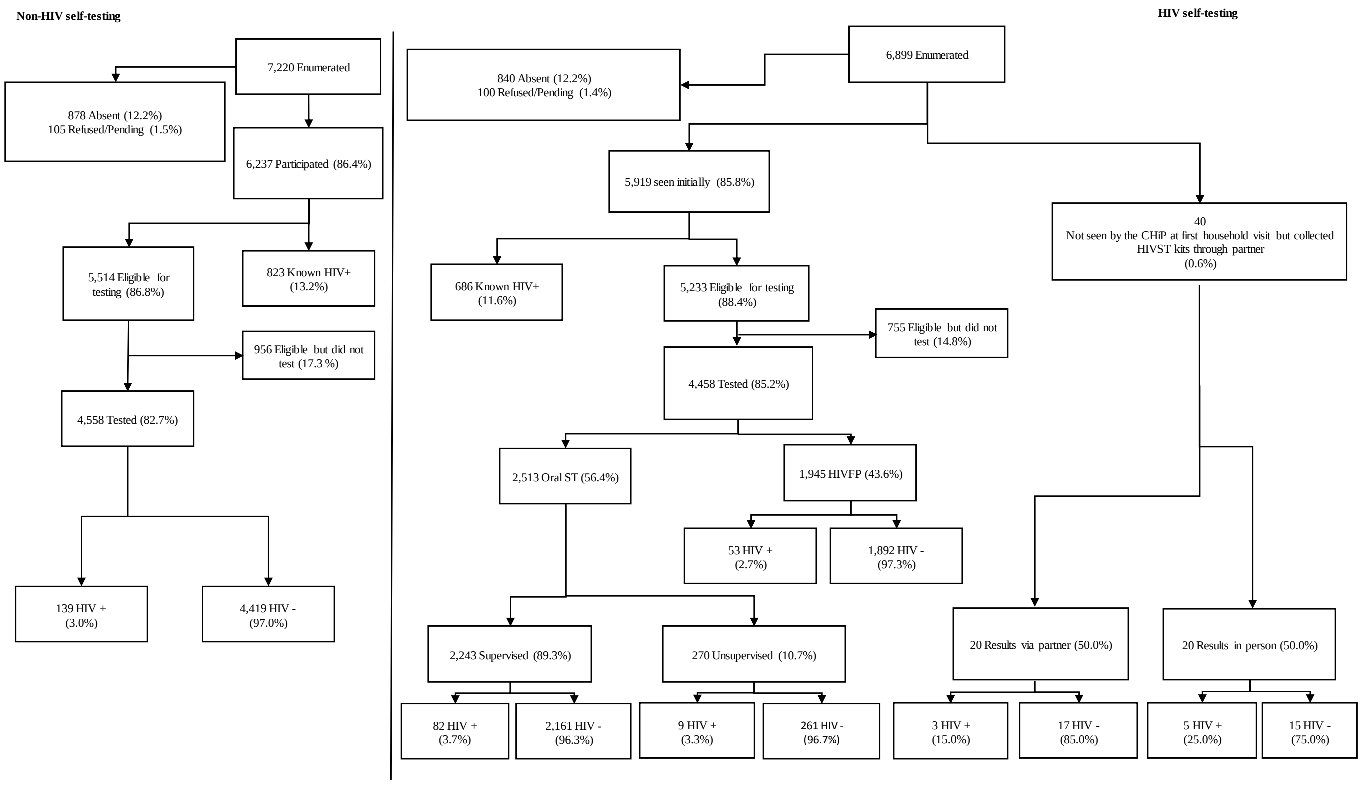

Appendix 6: Social harms matrix

Source 1	Source 2	Type
20170329_Z6_FGD	FGD (CHiPs)	Threatening harm/ divorce
20170329_Z6_FGD	FGD (CHiPs)	Couple separated
20170317_Z5_FGD	FGD (CHiPs)	Invasion of privacy
20170329_Z1_FGD	FGD (CHiPs)	Deceit / forced testing
20170321_Z6_IDI	Male (adolescent, accepted, supervised)	Deceit / forced testing
20170220_Z6_HIVST	FGD (CHiPs observation)	Forced testing
Monitoring visit	Case 1	Attempted forced testing
Monitoring visit	Case 3	Emotional distress
Monitoring visit	Case 4	Marriage Separation
Monitoring visit	Case 10	Emotional distress
Monitoring visit	Case 11	Emotional distress
Monitoring visit	Case 14	Blackmail (using results as evidence against spouse)
Monitoring visit	Case 17	GBV and Threatening suicide
**Key:** Z1, Z5 and Z6 are site codes		
